# Inhibitory interaction networks among coevolved *Streptomyces* populations from prairie soils

**DOI:** 10.1371/journal.pone.0223779

**Published:** 2019-10-31

**Authors:** Daniel C. Schlatter, Zewei Song, Patricia Vaz-Jauri, Linda L. Kinkel

**Affiliations:** 1 Department of Plant Pathology, University of Minnesota, Saint Paul, MN, United States of America; 2 Clement Estable Biological Research Institute, Avenida Italia, Montevideo, Uruguay; Nederlands Instituut voor Ecologie, NETHERLANDS

## Abstract

Soil microbes live within highly complex communities, where community composition, function, and evolution are the product of diverse interactions among community members. Analysis of the complex networks of interactions within communities has the potential to shed light on community stability, functioning, and evolution. However, we have little understanding of the variation in interaction networks among coevolved soil populations. We evaluated networks of antibiotic inhibitory interactions among sympatric *Streptomyces* communities from prairie soil. Inhibition networks differed significantly in key network characteristics from expectations under null models, largely reflecting variation among *Streptomyces* in the number of sympatric populations that they inhibited. Moreover, networks of inhibitory interactions within *Streptomyces* communities differed significantly from each other, suggesting unique network structures among soil communities from different locations. Analyses of tri-partite interactions (triads) showed that some triads were significantly over- or under- represented, and that communities differed in ‘preferred’ triads. These results suggest that local processes generate distinct structures among sympatric *Streptomyces* inhibition networks in soil. Understanding the properties of microbial interaction networks that generate competitive and functional capacities of soil communities will shed light on the ecological and coevolutionary history of sympatric populations, and provide a foundation for more effective management of inhibitory capacities of soil microbial communities.

## Introduction

Soil microbes exist in highly diverse communities in which thousands of microorganisms are estimated to coexist in a single gram of soil [1]. Competitive, antagonistic, cooperative, and syntrophic interactions among microbial species are crucial to maintaining microbial diversity, and significantly impact the dynamics, metabolic activities, and evolution of microbial populations [2–8]. However, despite their critical role in community ecology and functioning, we have very little insight into the structure of microbial interactions within coevolved soil communities. Most work to date examining microbial interactions has focused primarily on species pairs [3, 9–11], though there are some exceptions [12, 13]. In diverse soil communities interactions among species pairs can in turn impact other community members, resulting in intricate microbial interaction networks [5, 14]. This suggests that the outcomes of interactions among two species are likely to have cascading effects on other taxa, with significant impacts on community dynamics and emergent functions.

Network analyses have been applied throughout social, physical, and biological sciences and provide invaluable insight into the behavior of complex systems [15–18]. Network metrics provide a useful way to understand aggregate properties of a system and can offer key insights into the topological properties, hubs, and control points of a network [19, 20]. Additionally, network analyses can be used to identify functionally important sub-structures, or motifs, that occur at greater or lesser frequency than expected, to uncover the ‘design principles’ of a network [21, 22]. For example, this approach has successfully identified feed-forward loops in transcriptional networks [23] and ‘chain’ motifs in food webs [21, 24]. These simple sub-structures are believed to represent the building blocks of highly complex interaction networks [22].One important means of identifying the sub-structure components of a network is through triad analysis. Triads, or sets of three nodes, have proven to be important network structures because they can result in distinct relationships and dynamics among nodes beyond simple node pairs (dyads). For example, indirect connections among nodes may suggest hierarchical relationships among nodes [25]. As a result, triad censuses have been used to understand the tendency of networks to support social, hierarchical, or transitive relationships. Similarly, though motif analysis requires the use of null network models, analyses of observed network sub-structures can shed further light on the biology of complex systems [26].Analysis of graphlets, or induced network substructures, is not dependent on null network models and offers unique insights into the roles that particular nodes play across distinct networks [26].

Species interactions within complex microbial networks are often inferred through the analysis of marker gene co-occurrence patterns [27–30]. However, such analyses present two significant limitations. First, co-association patterns necessitate sampling across many different communities. Moreover, while co-occurrence patterns among taxa suggest the potential for interactions among co-existing taxa, they cannot shed light on the biological nature of these interactions. Enhanced understanding of the structure of species interactions within microbial communities will require explicit investigation of interactions among coevolved or co-occurring taxa from intact communities.

Co-evolution among species will shape networks of species interactions within a community. Thus, because species interaction networks will be a function of the ecological and coevolutionary history of a community, analyses of sympatric (co-occurring, or from the same community) but not allopatric (from different communities) populations are critical to understanding the innate structure of microbial interaction networks [3, 9, 11, 31]. In particular, artificially-composed networks among populations from diverse locations provide little insight into the community structures that result from natural assembly processes. Moreover, consideration of the specific network substructures that may be characteristic of individual isolates can provide an ‘interaction signature’ for a microbe and shed light on the ecology and coevolutionary biology of species interactions that structure microbial communities.

*Streptomyces* are Gram +, filamentous bacteria that are found ubiquitously in soils and sediments [32, 33]. *Streptomyces* are responsible for the production of more than 70% of the diverse array of clinical antibiotics derived from natural sources [34–36]. *Streptomyces* are also potent antagonists of plant pathogens and play important roles in plant disease suppression [32, 37–39]. Competitive and coevolutionary dynamics among *Streptomyces* are hypothesized to be critical to selection for antibiotic inhibitory and resistance phenotypes in soil [3, 9, 31]. In-depth characterization of the topology and motif structure of inhibition networks among sympatric populations is needed to provide insight into the landscape of interactions among coevolved *Streptomyces* populations, and on the dynamics of antibiotic inhibitory and resistant interactions in complex soil communities.

Here we use network analyses to explore the structure of inhibitory interactions among naturally-occurring, coevolved *Streptomyces* populations from prairie soils. Our objectives were to 1) characterize the structure of inhibition networks for three different communities of coevolved *Streptomyces*; 2) compare properties of *Streptomyces* inhibition networks among communities and with those of common network models; and 3) characterize the relative abundance of distinct three-member triads among coevolved *Streptomyces* communities. In total, these results shed light on the complex web of inhibitory interactions in naturally-coevolved *Streptomyces* communities and the variation in networks of inhibition among communities.

## Methods

### *Streptomyces* isolates and inhibition assay

*Streptomyces* were isolated from three soil cores at the Cedar Creek Ecosystem Science Reserve, a National Science Foundation Long-term Ecological Research Site, as described previously [40]. Fifty-two isolates *Streptomyces* (n = 17–18 isolates/individual soil core) were randomly chosen for characterization of inhibition phenotypes against each other. Isolate identity, core of origin, and Genbank accessions for previously obtained partial 16S rRNA gene sequences [41] are available in S1 Table.

Inhibitory interactions among all possible *Streptomyces* isolate pairs were evaluated using an agar-overlay method as described previously [40]. Briefly, for each interaction, 10ul of a *Streptomyces* spore suspension (~10^8^ cfu/ml) was spotted on the surface of a petri plate (15ml starch casein agar [SCA]), grown for 3 days, and killed by inverting over 4ml chloroform in a watch glass for 1h. After residual chloroform was allowed to evaporate, a fresh layer of 15ml SCA was overlaid on each plate and allowed to cool. Next, 100ul of a test isolate was spread plated and grown for 3 days. Zones of inhibition were measured twice at right angles and averaged for each measurement. Each *Streptomyces* isolate pair was replicated 3 times. Pairwise inhibition data among subsets of these isolates have been reported previously [3], yet no work to date has explored inhibition networks among these *Streptomyces* populations.

### *Streptomyces* inhibition networks

**Fig 1:**
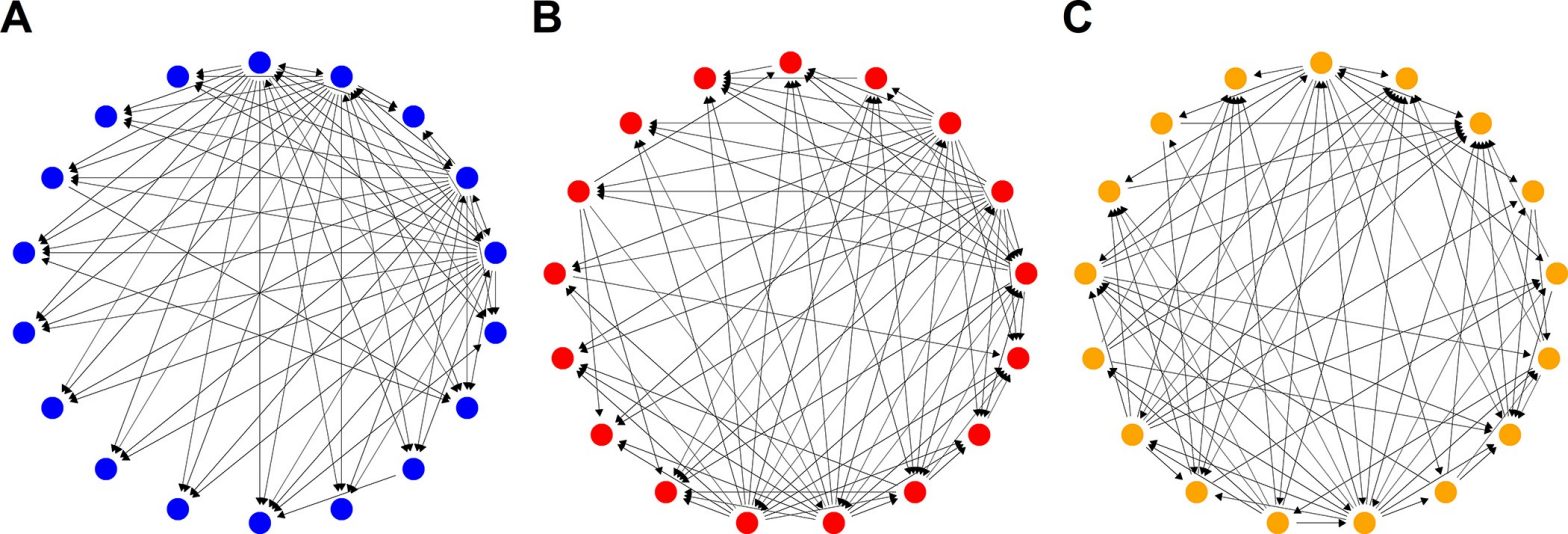
Networks of inhibitory interactions among *Streptomyces* isolates from different locations (designated ‘A’ [blue], ‘B’ [red], and ‘C’ [orange]). Nodes represent isolates whereas directed edges indicate antibiotic inhibitory interactions among isolates.

Inhibitory interactions among *Streptomyces* from the same soil core (sympatric) were treated as directed, unweighted networks and visualized in R [42](Fig 1, S2 Table). In these networks each node represents a randomly selected *Streptomyces* isolate and each edge represents inhibition by the source isolate against a target isolate. Edges were placed only where inhibition zones against a target isolate were larger than 2 mm. For each network the mean shortest path length [43] between nodes and the clustering coefficient (ie. the ratio of triangles to connected triples) were calculated [44] using the ‘igraph’ package in R [45]. Notably, shortest path lengths follow directed paths among nodes and will not include unconnected nodes or those lacking out-degrees. Metrics for each network were compared against null expectations based on 10,000 random networks generated using either an Erdős-Rényi (ER) model, or graphs ‘conditioned’ with the dyad frequencies or degree distributions of the original network. For ER networks, the same numbers of edges as in each of the original networks were placed randomly among the same number of nodes. For dyad-conditioned networks, networks were generated to have the same frequency of mutual, asymmetric, or null (no inhibition) inhibitory interactions among *Streptomyces* pairs as found in the original network. For network degree-conditioned models, the degree distribution of the original network was preserved by randomizing edges using an edge-swapping algorithm, where the original network is randomized by arbitrarily choosing two edges and swapping the in- and out-nodes of each edge, using the ‘rewire’ method in igraph [21](www.igraph.org). Edge-swapping was conducted 1000 times for each degree-conditioned network randomization. Mean shortest path lengths and clustering coefficients of *Streptomyces* interaction networks were compared with those evaluated for each of the 10,000 random networks using z-tests to assess the significance of differences of the observed networks from those of expectations under different network ‘null’ models [21].

### Triad motif analysis

Motifs are network substructures that occur significantly more or less frequently than expected by chance under null network models, and are often found to represent key functional structures in complex systems [22]. To identify 3-member interaction patterns (triads) that were significantly over- or under- represented in *Streptomyces* networks (triad motifs), triad frequencies were quantified for each empirical network and compared to frequencies of triads from modeled ‘null’ networks. For individual triads in each network, frequencies were compared those observed in 10,000 null networks using z-tests (described above). Triads were considered motifs if p-values were <0.05 after adjustment for multiple comparisons using an FDR correction [46]. In each network, overall triad frequencies were tested for significant departure from those expected in conditioned graphs with chi-squared goodness of fit tests using the mean frequency of triads in conditioned networks as the expected value. Pearson’s chi-squared test was used to compare the occurrence of triads among *Streptomyces* networks from different soil cores. Because the occurrence frequency of some triads was very low, p-values were computed using a Monte Carlo test using 2000 simulations for all chi-squared tests using the ‘coin’ package in R.

### Node participation

**Fig 2:**
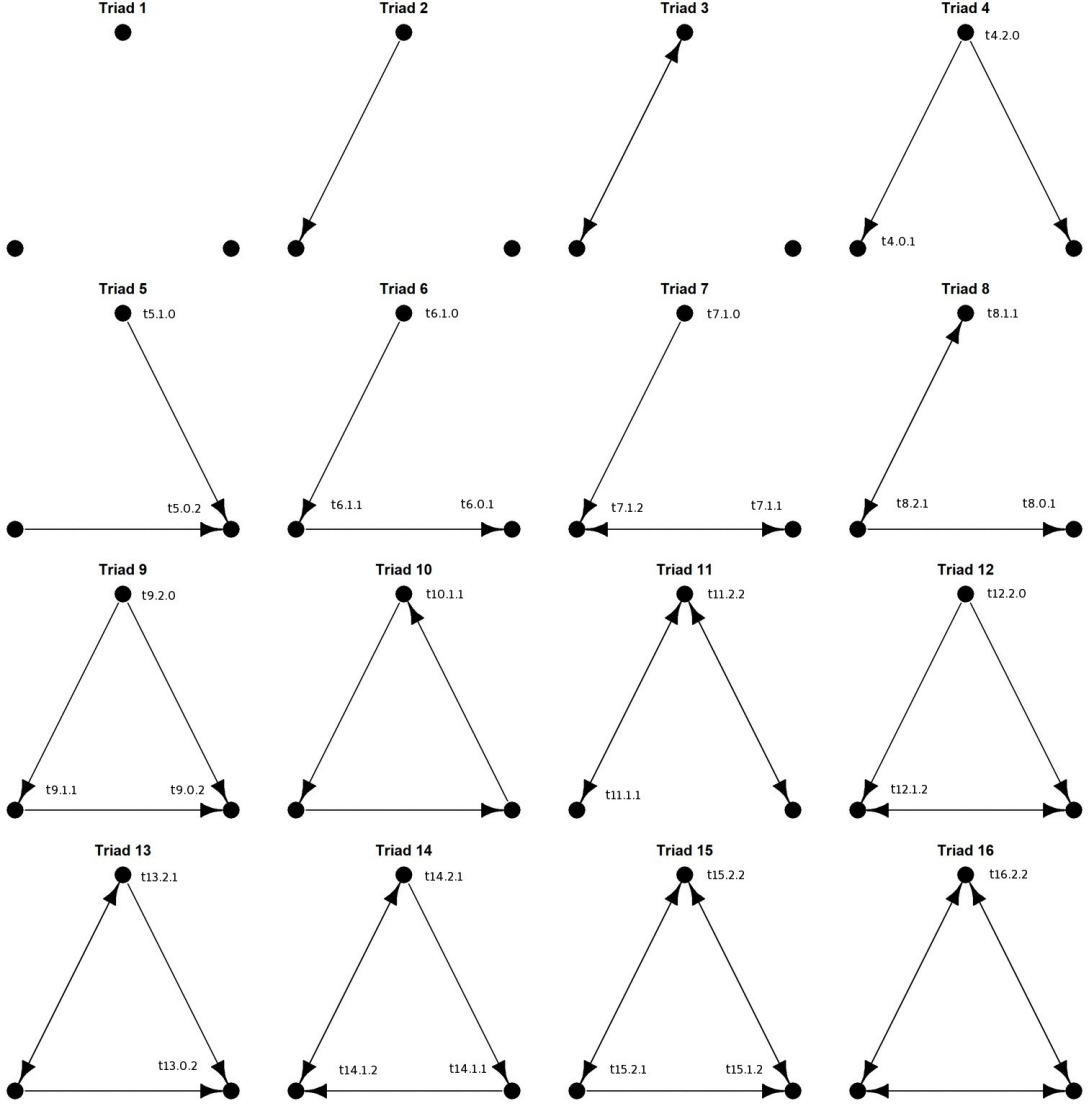
Structures of sixteen possible triads of directed interactions. Unique node positions within each connected triad are indicated with the triad number, out-degrees, and in-degrees for that node (eg. node t4.2.0 is within triad 4, has 2 out-degrees, and 0 in-degrees).

Analyses of node participation within realized network substructures (graphlets) offers an alternative approach to identifying important organizational patterns within and among networks [26, 47, 48]. To compare the participation of each *Streptomyces* isolate (node) in three-member triads within each community, we quantified the participation of each node in three-member interactions while taking in to account the position of each node. Isolates that hold similar positions are expected to play similar biological roles within their local communities. The frequency of node positions within each connected triad (for a total of 30 isomorphic node positions; Fig 2; [26] was used to generate a ‘signature vector’ for each node. The signature vector represents the cumulative local graph structure of each node (isolate). Similarity among *Streptomyces* in signature vectors was assessed with hierarchical clustering and Pearson correlations in R.

## Results

### *Streptomyces* inhibition networks

**Fig 3:**
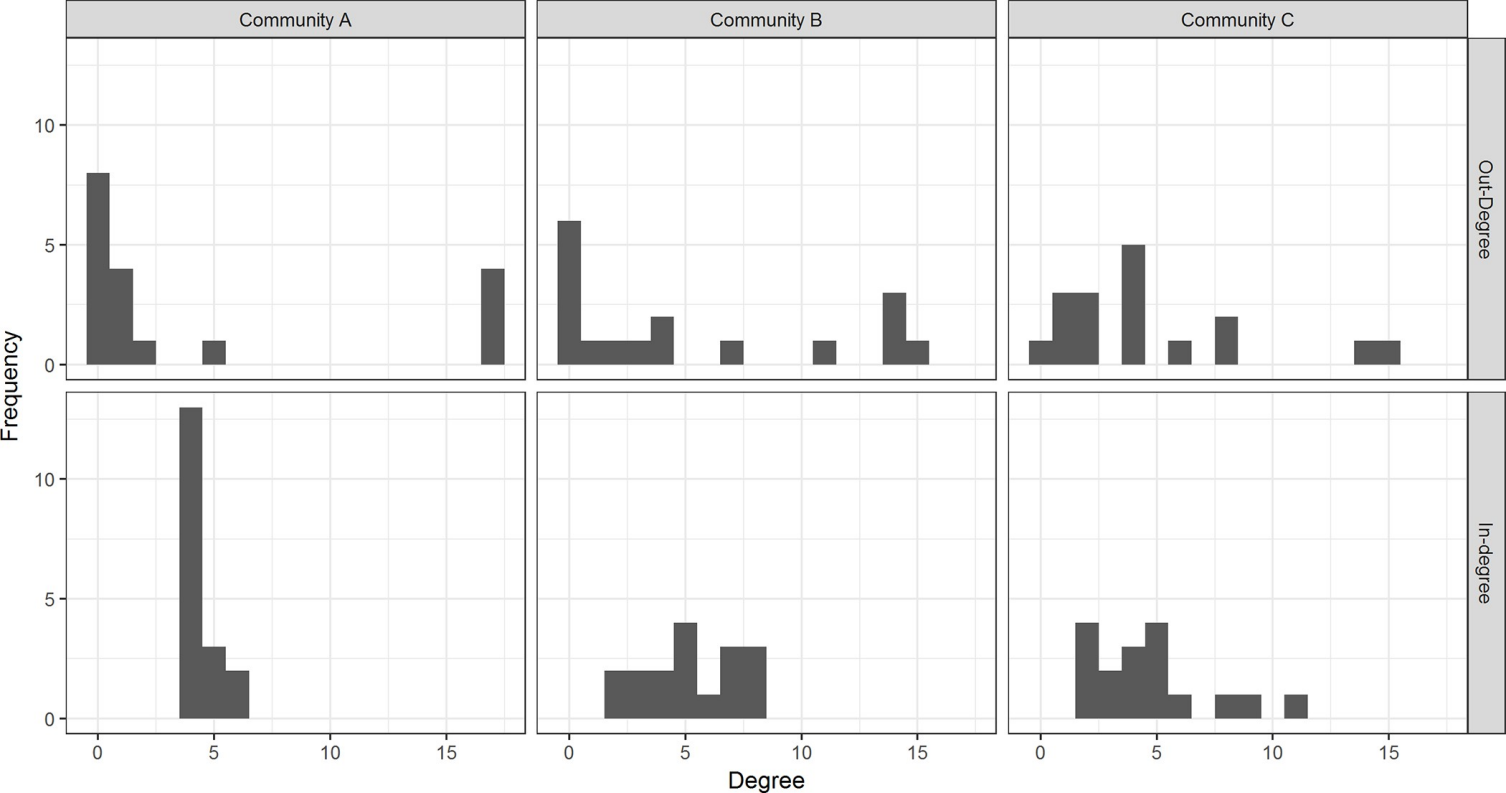
Distributions of out-degrees (upper) and in-degrees (lower) of network nodes from different locations.

Coevolved *Streptomyces* inhibition networks from the three different communities all exhibited complex patterns of highly-specific interactions among populations (Fig 1). The three communities varied in network structure, as captured in their distinct degree distributions (Fig 3). Although the density of the networks was similar (Table 1), the distribution of out-degrees among isolates, or the number of other isolates that each *Streptomyces* inhibited, varied notably among networks. The out-degree pattern was approximately bimodal for community A, indicating that *Streptomyces* from this community tended to either have very little or very strong capacities to inhibit other community members. In contrast, for community C most *Streptomyces* had moderate inhibitory capacities while there were very few highly inhibitory isolates or poor inhibitors. Community B had a more even out-degree distribution than either community A or C, with a greater number of poor inhibitors (small out-degree nodes). Thus, communities varied in the distribution of inhibitory capacities among isolates.

**Table 1:** Network metrics of real networks of coevolved Streptomyces inhibitory interactions and those of random network models.

Network Metric	Network A	Network B	Network C	Definition
**Number of Nodes**	18	17	17	Number of isolates
**Number of Edges**	79	89	80	Number of inhibitory interactions
**Graph Density**	0.258	0.327	0.294	Realized proportion of all possible edges among nodes
**Mean in-degree (+/- SD)**	4.39 +/- 0.70	5.24 +/- 2.05	4.71 +/- 2.59	Average number of isolates that a single isolate inhibits or is inhibited by
**Mean out-degree (+/- SD)**	4.39 +/- 7.04	5.24 +/- 5.93	4.71 +/- 4.36	Average number of isolates inhibited by a single isolate
**Mean Shortest Path (L)**	1.48	1.56	2.23	Average shortest graph distance between nodes
**L**_**degree-constrained**_ **(p-val)**	1.78 (0.13)	1.63 (0.37)	2.08 (0.51)	
**L**_**Erdos-Renyi**_ **(p-val)**	**2.00 (<0.0001)**	**1.79 (<0.0001)**	**1.89 (<0.0001)**	** **
**L**_**Dyad-constrained**_	**1.58 (<0.0001)**	**1.43 (p<0.0001)**	**1.47 (p<0.0001)**	** **
**Clustering Coeff (C)**	0.79	0.75	0.54	Ratio of triangles to connected triples.
**C**_**constrained**_ **(p-val)**	**0.76 (0.002)**	0.76 (0.52)	0.56 (0.37)	
**C**_**Erdos-Renyi**_ **(p-val)**	**0.25 (<0.0001)**	**0.32 (<0.0001)**	**0.29 (<0.0001)**	** **
**C**_**Dyad-constrained**_	**0.25 (<0.0001)**	**0.32 (<0.0001)**	**0.29 (<0.0001)**	** **
**Lambda**	0.95	1.07	1.48	Ratio of shortest path length to mean shortest path lengths in random ER graphs
**Gamma**	3.12	2.31	1.88	Ratio of clustering coefficient to mean clustering coefficient in random ER graphs
**Small-Worldness**	3.29	2.15	1.27	Lambda/Gamma as defined in Humphries and Gurney, 2008. Larger values indicate a greater degree of ‘small-world-ness’.

In a similar manner, the distribution of in-degrees, or the number of isolates each *Streptomyces* was inhibited by, differed among communities (Fig 3). In community A, most *Streptomyces* had small in-degrees indicating that individual isolates tended to be inhibited by very few other isolates; this is consistent with a substantial accumulation of antibiotic resistance across isolates. In-degrees in community network B exhibited a broader range of values, reflecting greater variation in resistance among populations. In community C, there was a higher frequency of isolates with large in-degrees than in community A or B, indicating that there are more *Streptomyces* that are susceptible to inhibition by many other isolates (less resistance).

Within all three communities, *Streptomyces* were connected by a small number of interactions, largely due to a small number of isolates within each community with a broad inhibition profile against other isolates (eg. isolates in community A: 1231.1, 1231.5, 1231.6; community B: 3211.6, 3211.5, 3211.3; Community C: 5111.1, 5112.6). Mean shortest path lengths, or the average shortest distance (number of interaction links) between individual *Streptomyces* pairs that can be connected via directed interaction links, tended to be very short, with an average of 1.5 to 2.2 interactions between isolates. These shortest path lengths within each community differed significantly from those expected from ER or dyad-conditioned network models. Specifically, path lengths observed in communities A and B were significantly smaller than among ER or dyad-conditioned networks (Table 1). In contrast, community C had a significantly greater mean shortest path length than expected from the ER or dyad-conditioned models. However, mean shortest path lengths of none of the networks A, B, or C differed significantly from those expected under the degree-conditioned network models (Table 1). Thus, the distinct distributions of within-community pairwise inhibitory interactions are critical determinants of inter-connectedness as characterized by path length within *Streptomyces* networks. In particular, small mean shortest path lengths correspond with a relatively high frequency of ‘super-killers’ (isolates having high out-degrees) that serve as hubs to connect many isolates within the community.

The clustering coefficient of each network, or the tendency for inhibitory interactions between *Streptomyces* within communities to occur among sub-groups of isolates, similarly varied among communities. Communities with shorter mean path lengths (communities A and B) had larger clustering coefficients than those with longer mean path lengths (community C). Clustering coefficients were significantly greater for the observed community network than for the corresponding ER or dyad-conditioned network models (Table 1). However, clustering in observed *Streptomyces* communities was only significantly greater from that of degree-conditioned network models for community A, and did not differ from degree-conditioned network models for communities B or C (Table 1), suggesting that degree distributions are sufficient to account for clustering within communities B and C.

### Triad motif analysis

**Fig 4:**
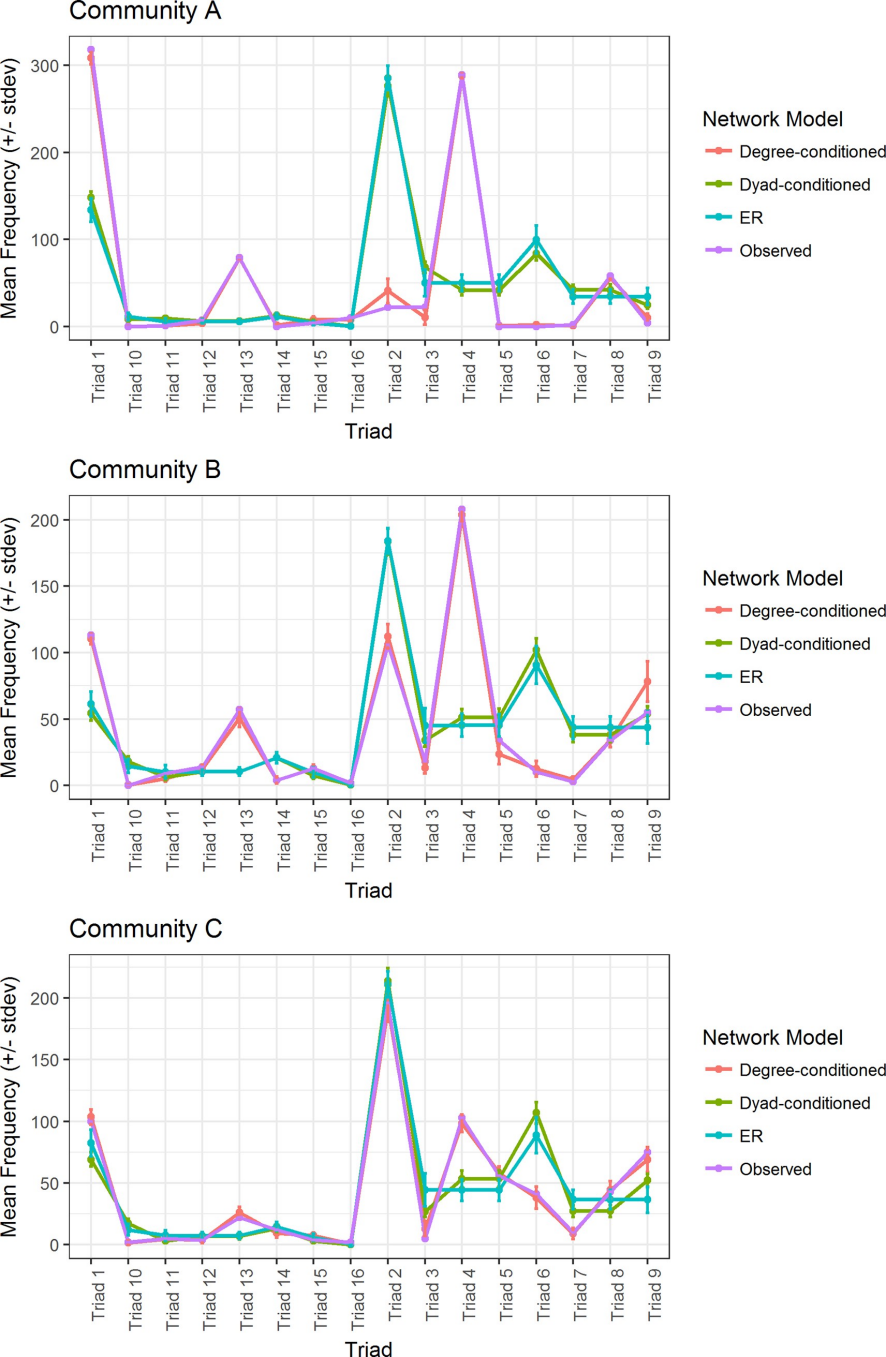
Frequencies of individual triads in each community compared with random network models.

We further explored the structure of *Streptomyces* inhibition networks by characterizing the frequency of distinct, 3-member triads within each community. There are 16 possible interaction triads in directed networks (Fig 3). Six triad structures (triads 1, 2, 3, 8, 9, and 13) were common across all three *Streptomyces* communities, whereas other triads were absent or occurred very rarely (eg. triads 7, 10, 11, 14, and 16) (Fig 4). Not all triads were observed in each community. In all three communities, the overall frequencies of distinct triads in *Streptomyces* inhibition networks differed significantly from expected frequencies under both random ER and dyad-conditioned null models (Chi-squared ≥70.1, p<0.00001 in each case). However, in networks A and C, triad relative frequencies did not differ significantly from expected values under the degree-conditioned model (Chi-squared≥12.1, p≥0.66). Thus, under the more stringent constraint in which expected triad relative abundances are conditioned on the observed distributions of in- and out-degrees among isolates, triad abundances in communities A and C are predicted by degree distributions. In contrast, triad relative frequencies in network B differed significantly from expected values under the degree-constrained model (Chi-squared = 71.3, p<0.0001). These results indicate that the frequencies of different triads in all three communities were non-random, but when accounting for the network degree distribution only community B had overall triad frequencies that were significantly different than expected. This suggests that triad frequencies within *Streptomyces* inhibitory networks are not necessarily explained only by the distribution of inhibitory phenotypes among isolates.

Consideration of the relative frequency of individual triads within and among communities provides further insights into the potential variation in ecological and coevolutionary dynamics across communities. When comparing triad frequencies among communities, the frequencies of specific triads varied significantly among communities (Fig 4; Pearson’s chi-squared = 634, p<0.0001). However, considering ER or dyad-conditioned network models, motifs 4 and 13 were significantly over-represented in all three communities, while motifs 6 and 10 were significantly under-represented. In contrast, when utilizing the degree-conditioned model, there were no triads that were consistently significantly over- or under-represented across the three communities (Table 2) Instead, triad motifs were generally unique to that community. For example, although both communities A and B had triads that were significantly over- or under-represented, compared to degree-conditioned network models (community A: triads 1, 2, 3, 7, 8, 9, 12, 15; community B: triads 5, 9, 11), no triads in community C were significantly enriched or diminished. When comparing triad frequencies among observed networks, the frequencies of specific triads varied significantly among *Streptomyces* communities from different locations (Fig 3; Pearson’s chi-squared = 634, p<0.0001). Additionally, frequencies of each individual triad differed significantly among networks (Pearson’s chi-squared>6.3, p<0.05), with the exception of triad 10 (Pearson’s chi-squared = 4, p = 0.14), which was found only in community C and occurred only twice.

**Table 2:** Comparison of triad frequencies in empirical versus degree-conditioned random networks. P-values are based on z-tests comparing the empirical frequency of each triad to the expected value based on mean frequency in conditioned networks.

**Network A**	Triad 1	Triad 2	Triad 3	Triad 4	Triad 5	Triad 6	Triad 7	Triad 8	Triad 9	Triad 10	Triad 11	Triad 12	Triad 13	Triad 14	Triad 15	Triad 16
Empirical Frequency	318	22	22	289	0	0	2	58	4	0	1	7	79	0	4	10
Mean Frequency Conditioned	302	53.097	2.9142	288.03	1.0325	2.0192	0.277	54.343	13.476	0.0695	0.6293	1.015	76.948	2.7317	11.361	6.051
p-value (FDR-adjusted)	**0.0021**	**0.0021**	**0.0021**	0.7154	0.2062	0.2175	**0.0053**	**0.0405**	**0.0176**	0.7862	0.5061	**0.0032**	0.2415	0.0563	**0.018**	**0.0104**
**Network B**	Triad 1	Triad 2	Triad 3	Triad 4	Triad 5	Triad 6	Triad 7	Triad 8	Triad 9	Triad 10	Triad 11	Triad 12	Triad 13	Triad 14	Triad 15	Triad 16
Empirical Frequency	113	106	18	208	34	10	3	34	55	0	9	14	57	4	13	2
Mean Frequency Conditioned	108.22	113.79	8.5286	202.24	16.284	18.654	4.9684	30.991	96.145	0.4075	2.8139	11.678	47.704	5.4739	11.083	1.0295
p-value (FDR-adjusted)	0.546	0.5798	0.087	0.4991	**0.0217**	0.4219	0.5674	0.6174	**0.0167**	0.6174	**0.0135**	0.5798	0.4219	0.6174	0.5798	0.4991
**Network C**	Triad 1	Triad 2	Triad 3	Triad 4	Triad 5	Triad 6	Triad 7	Triad 8	Triad 9	Triad 10	Triad 11	Triad 12	Triad 13	Triad 14	Triad 15	Triad 16
Empirical Frequency	100	196	5	103	56	41	10	43	75	2	5	4	22	12	4	2
Mean Frequency Conditioned	103.47	193.18	13.538	96.844	57.907	37.478	9.0169	44.989	68.395	1.7265	5.0763	4.1212	27.213	8.7982	7.3634	0.8747
p-value (FDR-adjusted)	0.9727	0.9727	0.9693	0.9727	0.9727	0.9727	0.9727	0.9727	0.9727	0.9727	0.9727	0.9727	0.9693	0.9727	0.9693	0.9693

### *Streptomyces* node participation

**Fig 5:**
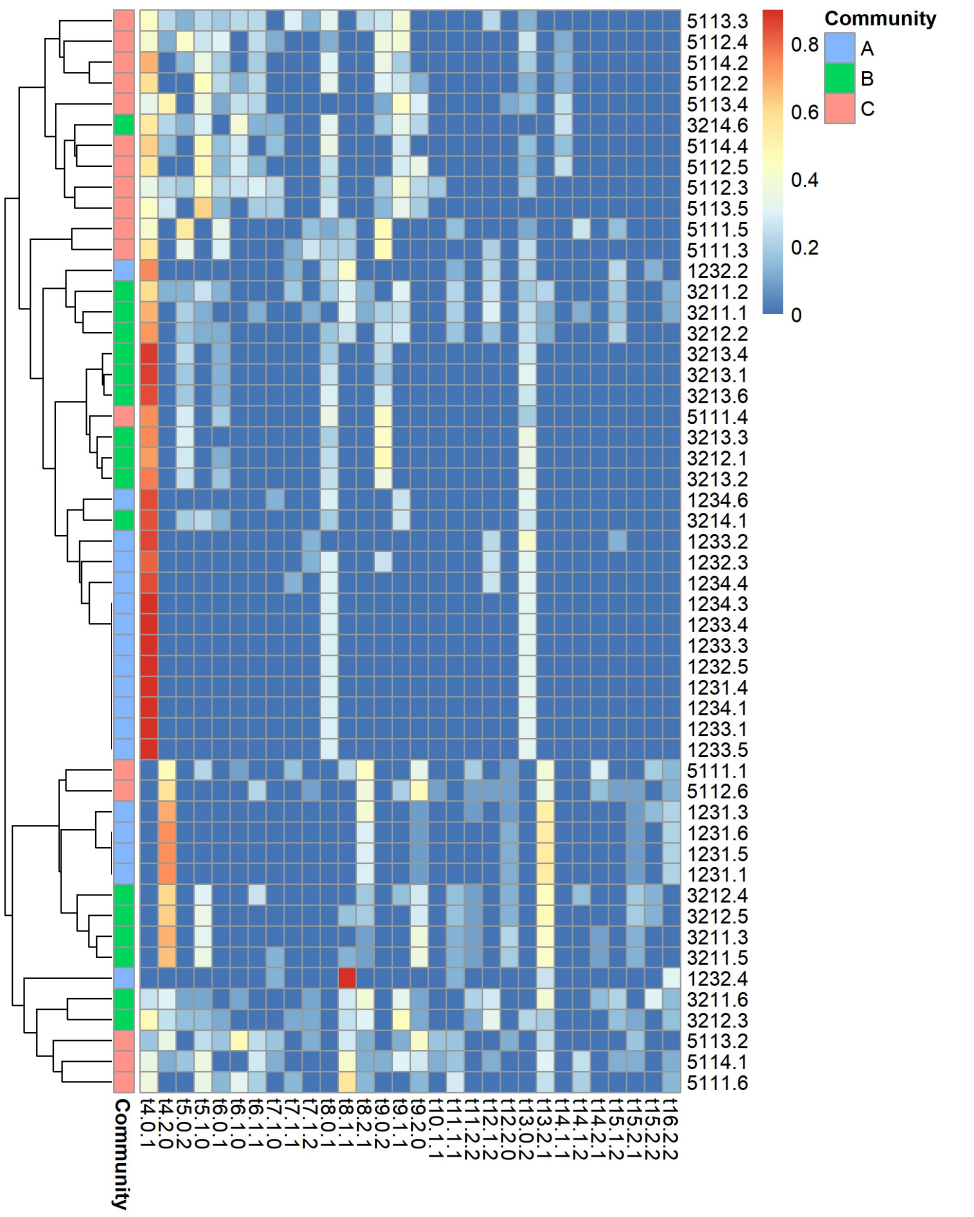
Heatmap square-root-transformed proportions of node participation in connected triads among nodes (right panel). Node participation allows for the comparison of the local structure of nodes among different networks. Labels indicate the triad (eg. t4 is triad 4), where the numbers following the triad identifier indicate the in- and out-degrees of the node within that triad.

Quantifying node (*Streptomyces* isolate) positions within triads revealed similarities in the local interaction structure of individual *Streptomyces* isolates (Fig 5). *Streptomyces* clustered into six groups that had similar local network structure. These groups were characterized by high frequencies of four node positions within 2 triads (positions triad 4:0,1, triad 4:2,0; triad 5:1,0; triad 8:1,1; triad 8: 2,1; triad 13:2,1; triad 13:0,2). Some of these clusters were dominated by *Streptomyces* from a particular community (soil core), indicating that these isolates interacted in unique ways within their local community, whereas other clusters with similar node participation consisted of isolates from all three communities. Moreover, the diversity of node participation varied significantly among communities (ANOVA F = 41.22, p<0.0001), and was lowest in community A (Shannon H’ = 0.89 +/- 0.30), intermediate in community B (Shannon H’ = 1.65 +/- 0.53), and highest in community C (Shannon H’ = 2.06 +/- 0.32). Thus some interaction patterns tended to be community-specific, whereas others occurred frequently among all communities. Variation in functioning among communities may be related to the presence or frequency of *Streptomyces* with particular local interaction structures.

## Discussion

Soil microbial communities are complex, dynamic systems where network analyses will be critical for unraveling the intricate structure of microbial interactions in soil. Networks of co-associations of genetic marker sequences are now commonly used to infer microbial interactions [27], yet the biological bases of co-associations are rarely validated and the capacities of co-associations in sequence abundances across many communities to shed light on the species interaction structure within communities remains limited. Networks of phenotypic interactions among microbial taxa offer a much clearer picture of interactions within intact microbial communities. Here we show that among soil *Streptomyces* communities, networks of inhibitory interactions within coevolved communities exhibited numerous characteristics distinct from those of common network models and from each other, and suggest key features that organize community-wide patterns of inhibitory interaction networks.

*Streptomyces* inhibition tended to occur more frequently among subgroups of isolates within communities than in ER or dyad-conditioned models. This clustering of interactions may have important implications for the co-evolutionary dynamics of antibiotic inhibitory and resistance traits. It has been observed previously that pairwise inhibition among *Streptomyces* is enriched for mutual inhibition, which is suggested to contribute to the maintenance of community stability, diversity, and evolution [9, 14]. However, since the costs or benefits of inhibitory interactions between *Streptomyces* pairs may be impacted by interactions with a third species, higher-order interactions among taxa are likely to influence selection for antibiotic production phenotypes. Thus, interactions among relatively small subsets of taxa may play a disproportionate role in microbial community dynamics and contribute to the antibiotic inhibitory and resistance potential of soil communities [49, 50]. Highly interactive clusters may represent sets of *Streptomyces* that share similar niches and compete strongly for resources that then rely on antibiotics as a means of defense. In this case, deployment of antibiotics among members of these clusters may play an especially key role in community assembly and the maintenance of community structure [51].

Path lengths, or the number of inhibitory interactions between any *Streptomyces* pair within networks, were very small, again highlighting the potential for beyond-pairwise interactions to be crucial for *Streptomyces* fitness. Specifically, because of the high interconnectivity of isolates, interactions between one pair of isolates are likely to have indirect effects on many other isolates. The high clustering and small path lengths of *Streptomyces* inhibition networks suggest that they have small-world characteristics, similar to many other biological systems [19]. It is hypothesized that evolution may favor biological systems with small-world characteristics due to their robustness in the face of disturbance [52]. Thus, the small path-length networks of inhibitory interactions among *Streptomyces* communities may contribute to community stability in response to invading populations, mutations, phage, or physical disruption.

Many of the non-random characteristics of *Streptomyces* inhibitory networks could be explained by network degree distributions, or the distribution of inhibition and resistance capacities among members of each community. Notably, a key feature of the degree distributions of inhibition networks was that each community harbored many isolates with little or no inhibitory capacity and a small number of highly inhibitory ‘super-killers’ that had the capacity to inhibit many other members of the community. This bimodal pattern has been observed in other studies [9, 40, 53]. Because of their highly inhibitory nature, these super-killer isolates may be keystones that play a significant role in controlling microbial community dynamics in soil ecosystems. However, despite the presence of super-killers in every community, their lack of complete dominance suggests the existence of factors limiting their fitness within the communities. For example, fitness costs of antibiotic production or resistance [54, 55] or inter-species signaling [11, 56] are likely to mediate the competitive outcomes of *Streptomyces* interactions.

**Fig 6:**
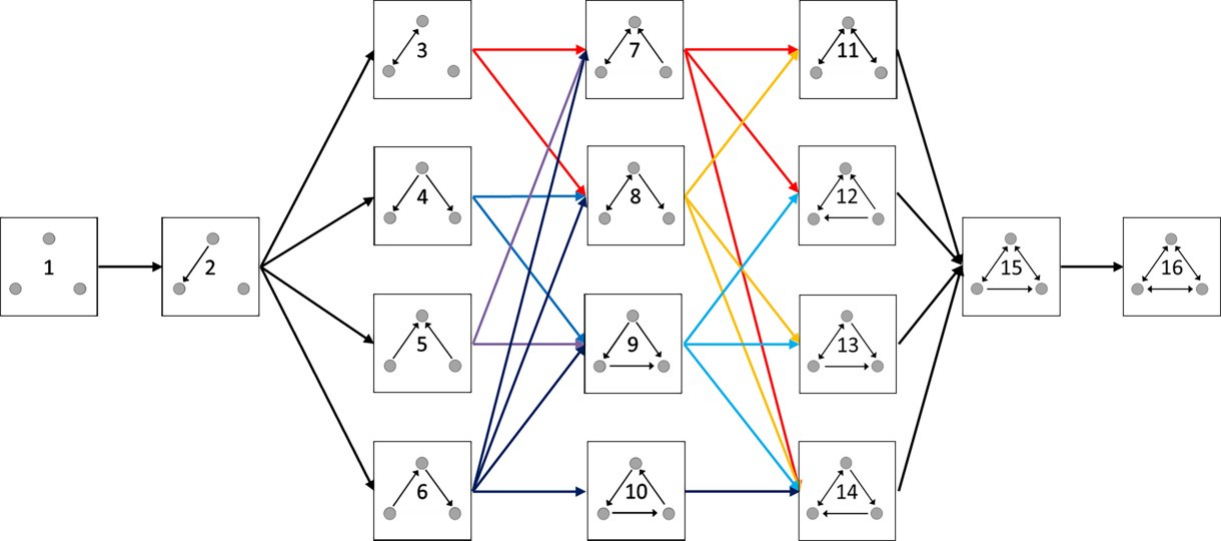
Conceptual figure of potential step-wise triad transitions, where transition is characterized by the gain of an antibiotic phenotype that specifically inhibits one other triad member or the loss of an antibiotic resistance phenotype. These transitions may occur as a result of demographic changes in communities or evolutionary processes.

Decomposing interaction networks into triads revealed that communities had rich triad structures that differed from common network models and varied among communities. Intriguingly, *Streptomyces* triads 4 and 13 were over-represented motifs across all communities, while triads 6 and 10 were under-represented motifs across all three communities. Further consideration of the characteristics of these motifs across communities may shed light on selection pressures likely to result in common or rare triads. For example, considering step-wise accumulation of resistance or inhibitory phenotypes (Fig 6), among possible triads having the same total number of inhibitory interactions, our data suggest both that those triads with the smallest number of required antibiotics, and with the least accumulated resistance will be relatively more common, while those requiring more antibiotics and resistance will be significantly less likely. At the scale of individual isolates, this suggests selection for broad-spectrum or multi-target antibiotics, and selection against accumulating specific resistance. Previous work has shown that resistance and inhibitory phenotypes impose fitness costs on microbes [57], so that consideration of both the costs and benefits of inhibitory and resistance phenotypes within networks will be critical to understanding the network structure of communities. Of note, triad 10, which represents the well-studied ‘rock-paper-scissors’ structure, and has been argued to be evolutionarily stable [58], was nearly absent from all empirical networks. Thus, despite its apparent evolutionary stability in experimental and modeling systems, the requirement for highly specific antibiotic inhibitory and resistance phenotypes in the rock-paper-scissors triad or dispersal effects [59] may make it an uncommon dynamic within *Streptomyces* communities in soil.

Though networks from different locations shared some common motifs versus simple network models, each community had motifs unique to that location compared to degree-conditioned models. We suggest that significant enrichment or diminishment of triads as compared with the degree-constrained model predictions reflects the selection on three-way interactions within the community. In particular, the significant non-random patterns of triad relative abundance in communities A and B suggest significant ongoing arms race coevolutionary dynamics in which specific triads are selectively enriched. This may be a function of transitory triads that reflect recent acquisition of inhibitory or resistance phenotypes in an ongoing arms race (e.g. transitions from triad 3 to 7 or 8, triad 7 to 11, 12, or 14; Fig 6). Alternatively this may provide evidence that selection acts on higher-order, three-way isolate combinations to selectively enrich or diminish particular triads. In contrast, community C, in which individual triad frequencies are not different from the degree-constrained predictions, may be at equilibrium with little ongoing selection within individual pairwise interactions (non-transitory/more stable), or lack of higher-order selection on triads. Moreover, the variation in motifs among communities suggests that the significance of specific interaction structures is likely to be context-dependent. For example, the particular motifs selected for or against in any given location may depend on the physical environment (pH, moisture, resource availability, etc), biological characteristics (microbial densities, community composition, etc), or historical processes (dispersal, disturbance, etc) [57–59].

Differential triad frequencies among communities may also reflect the temporal state or evolutionary history of that community. Specifically, the higher-order structure of inhibition networks may be temporally dynamic due to demographic changes in populations and the evolution of antibiotic inhibitory or resistance phenotypes among constituent members. Potential transitions between triad structures (Fig 6) may result from fluctuating populations, as some members of triads out-compete others, or the evolutionary gain or loss of antibiotic production or resistance by individual nodes. Notably, unless a new node (population) participates in the interaction network, the creation of one triad will result in the destruction of another. Future work exploring the temporal dynamics of *Streptomyces* interaction networks will provide insights into the evolution of network structure, the dynamic inter-relationships among network sub-structures, and the role that selection plays in determining the phenotypic composition of communities within a network context.

Comparing node participation signature vectors of *Streptomyces* from co-evolved communities revealed that individual *Streptomyces* were grouped into only a few clusters with similar positions in triads, suggesting that members of these groups play similar functional roles within their co-evolved inhibitory networks. However, many of the clusters of similar node participation contained *Streptomyces* that originated from only one community, which may reflect unique selection pressures for specific species interaction structures within communities. In contrast, one cluster contained *Streptomyces* from all communities, indicating that some *Streptomyces* may play similar roles among distinct communities.

There are numerous practical implications for understanding the network structure of inhibitory interactions among *Streptomyces* in soil. For example, enhancing the densities and antibiotic inhibitory phenotypes of *Streptomyces* populations in soil for plant disease suppression has been hypothesized as a means of pathogen management in agricultural systems [60–62]. This suggests that ‘super-killer’ isolates that serve as keystone or hub taxa are promising candidates for targeted manipulation of community functions [63–64]. However, differences in the degree distributions of inhibitory phenotypes among communities, as observed between the communities evaluated here and around the globe [53], communities are likely to vary in their robustness to change, which may contribute to the large variation observed in the success of biocontrol organisms and management practices [65]. This suggests that if we seek to manipulate interactions among soil community members we must first identify the important ‘control points’ of the system. Manipulating the densities, frequencies, or activities of the keystone or ‘control point’ taxa, either through inoculation or disruption of key populations or by altering resource or environmental conditions to modify their densities or behaviors, may offer a means for rational management of microbial interactions for targeted functional outcomes. These approaches could be used as a complement to traditional biocontrol efforts, where disruption of inhibitory networks may directly or indirectly facilitate the establishment of introduced biocontrol organisms that would otherwise be unable to colonize.

Network analyses continue to provide novel insights into complex biological systems. Although increasingly used to study co-occurrence patterns of microbial taxa, complementing these studies with detailed examination of phenotypic interactions within microbial communities will be crucial to revealing the principles that underlie community functions related to microbial species interactions. Future work using experimental communities will shed light on the temporal dynamics and evolutionary significance of interaction motifs within multi-species networks and could be used to inform mathematical models of species interactions [66]Moreover, considering larger networks of microbial interactions encompassing a more diverse range of taxa and phenotypes, such as resource use [67], inter-species signaling [56], and metabolite exchange [68], and considering them as interconnected, multilayered networks [69] will provide a much more nuanced and holistic understanding of the complex inter-relationships among soil microbes.

## Supporting information

S1 Table *Streptomyces* isolates obtained from each community (soil core), genbank accession numbers for partial 16S rRNA gene sequences, and RDP classification to genus. (XLSX)

S2 Table Interaction matrix among Streptomyces isolates for communities A, B, and C. Ones represent inhibitory phenotypes of the isolates in the row against isolates in the columns.
(XLSX)

## References

[1] Torsvik V, Øvreås L (2002) Microbial diversity and function in soil: from genes to ecosystems. Curr Opin Microbiol 5:240–245 12057676 10.1016/s1369-5274(02)00324-7

[2] Czaran TL (2002) Chemical warfare between microbes promotes biodiversity. Proc Natl Acad Sci 99:786–790. 10.1073/pnas.012399899 11792831 PMC117383

[3] Kinkel LL, Schlatter DC, Xiao K, Baines AD (2014) Sympatric inhibition and niche differentiation suggest alternative coevolutionary trajectories among Streptomycetes. ISME J 8:249–256. 10.1038/ismej.2013.175 24152720 PMC3906824

[4] Cordero OX, Wildschutte H, Kirkup B, Proehl S. Ngo L, Hussain F, et al (2012) Ecological Populations of Bacteria Act as Socially Cohesive Units of Antibiotic Production and Resistance. Science 337:1228–1231. 10.1126/science.1219385 22955834

[5] Lawrence D, Fiegna F, Behrends V, Bundy JG, Phillimore AB, Bell T, et al (2012) Species Interactions Alter Evolutionary Responses to a Novel Environment. PLoS Biol 10:e1001330 10.1371/journal.pbio.1001330 22615541 PMC3352820

[6] Fiegna F, Moreno-Letelier A, Bell T, Barraclough TG (2015) Evolution of species interactions determines microbial community productivity in new environments. ISME J 9:1235–1245. 10.1038/ismej.2014.215 25387206 PMC4409166

[7] Traxler MF, Watrous JD, Alexandrov T, Dorrestein PC, Kolter R (2013) Interspecies Interactions Stimulate Diversification of the Streptomyces coelicolor Secreted Metabolome. mBio 4:e00459-13–e00459-13. 10.1128/mBio.00459-13 23963177 PMC3747584

[8] Harrison E, Hall JPJ, Paterson S, Spiers AJ, Brockhurst MA (2017) Conflicting selection alters the trajectory of molecular evolution in a tripartite bacteria-plasmid-phage interaction. Mol Ecol 26:2757–2764. 10.1111/mec.14080 28247474 PMC5655702

[9] Vetsigian K, Jajoo R, Kishony R (2011) Structure and Evolution of Streptomyces Interaction Networks in Soil and In Silico. PLoS Biol 9:e1001184 10.1371/journal.pbio.1001184 22039352 PMC3201933

[10] Slattery M, Rajbhandari I, Wesson K (2001) Competition-Mediated Antibiotic Induction in the Marine Bacterium Streptomyces tenjimariensis. Microb Ecol 41:90–96. 10.1007/s002480000084 12032613

[11] Vaz Jauri P, Kinkel LL (2014) Nutrient overlap, genetic relatedness and spatial origin influence interaction-mediated shifts in inhibitory phenotype among *Streptomyces* spp. FEMS Microbiol Ecol 90:264–275. 10.1111/1574-6941.12389 25098381

[12] Pérez-Gutiérrez R-A, López-Ramírez V, Islas Á, Alcaraz LD, Hernandez-Gonzalez I, Olivera BC, et al (2013) Antagonism influences assembly of a Bacillus guild in a local community and is depicted as a food-chain network. ISME J 7:487–497. 10.1038/ismej.2012.119 23096405 PMC3578566

[13] Prasad S, Manasa P, Buddhi S, Singh SM, Shivaji S (2011) Antagonistic interaction networks among bacteria from a cold soil environment. FEMS Microbiol Ecol 78:376–385. 10.1111/j.1574-6941.2011.01171.x 22092175

[14] Kelsic ED, Zhao J, Vetsigian K, Kishony R (2015) Counteraction of antibiotic production and degradation stabilizes microbial communities. Nature 521:516–519. 10.1038/nature14485 25992546 PMC4551410

[15] Gurney J, Aldakak L, Betts A, Gougat-Barbera C, Poisot T, Kaltz O, et al (2017) Network structure and local adaptation in co-evolving bacteria-phage interactions. Mol Ecol 26:1764–1777. 10.1111/mec.14008 28092408

[16] Kurvers RHJM, Krause J, Croft DP, Wilson ADM, Wolf M (2014) The evolutionary and ecological consequences of animal social networks: emerging issues. Trends Ecol Evol 29:326–335. 10.1016/j.tree.2014.04.002 24792356

[17] Pinter-Wollman N, Hobson EA, Smith JE, Edelman J, Shizuka D, de Silva A, et al (2014) The dynamics of animal social networks: analytical, conceptual, and theoretical advances. Behav Ecol 25:242–255. 10.1093/beheco/art047

[18] Proulx S, Promislow D, Phillips P (2005) Network thinking in ecology and evolution. Trends Ecol Evol 20:345–353. 10.1016/j.tree.2005.04.004 16701391

[19] Watts DJ, Strogatz SH (1998) Collective dynamics of “small-world” networks. Nature 393:440–442. 10.1038/30918 9623998

[20] Dubitzky W, Wolkenhauer O, Cho K-H, Yokota H (2013) Encyclopedia of systems biology. Springer Reference, New York

[21] Milo R (2002) Network Motifs: Simple Building Blocks of Complex Networks. Science 298:824–827. 10.1126/science.298.5594.824 12399590

[22] Alon U (2007) Network motifs: theory and experimental approaches. Nat Rev Genet 8:450–461. 10.1038/nrg2102 17510665

[23] Mangan S, Alon U (2003) Structure and function of the feed-forward loop network motif. Proc Natl Acad Sci 100:11980–11985. 10.1073/pnas.2133841100 14530388 PMC218699

[24] Stouffer DB (2010) Scaling from individuals to networks in food webs. Funct Ecol 24:44–51. 10.1111/j.1365-2435.2009.01644.x

[25] Shizuka D, McDonald DB (2015) The network motif architecture of dominance hierarchies. J R Soc Interface 12:. 10.1098/rsif.2015.0080 PMC4387537 25762649

[26] Trpevski I, Dimitrova T, Boshkovski T, Stikov N, Kocarev L (2016) Graphlet characteristics in directed networks. Sci Rep 6:. 10.1038/srep37057 PMC5103263 27830769

[27] Poudel R, Jumpponen A, Schlatter DC, Paulitz TC, Gardener BB, Kinkel LL, et al (2016) Microbiome Networks: A Systems Framework for Identifying Candidate Microbial Assemblages for Disease Management. Phytopathology 106:1083–1096. 10.1094/PHYTO-02-16-0058-FI 27482625

[28] Barberán A, Bates ST, Casamayor EO, Fierer N (2011) Using network analysis to explore co-occurrence patterns in soil microbial communities. ISME J 6:343–351. 10.1038/ismej.2011.119 21900968 PMC3260507

[29] Bakker MG, Schlatter DC, Otto-Hanson L, Kinkel LL (2014) Diffuse symbioses: roles of plant-plant, plant-microbe and microbe-microbe interactions in structuring the soil microbiome. Mol Ecol 23:1571–1583. 10.1111/mec.12571 24148029

[30] Layeghifard M, Hwang DM, Guttman DS (2017) Disentangling Interactions in the Microbiome: A Network Perspective. Trends Microbiol 25:217–228. 10.1016/j.tim.2016.11.008 27916383 PMC7172547

[31] Essarioui A, Kistler HC, Kinkel LL (2016) Nutrient use preferences among soil Streptomyces suggest greater resource competition in monoculture than polyculture plant communities. Plant Soil 409:329–343. 10.1007/s11104-016-2968-0

[32] Kinkel LL, Schlatter DC, Bakker MG, Arenz BE (2012) Streptomyces competition and co-evolution in relation to plant disease suppression. Res Microbiol 163:490–499. 10.1016/j.resmic.2012.07.005 22922402

[33] Seipke RF, Kaltenpoth M, Hutchings MI (2012) Streptomyces as symbionts: an emerging and widespread theme? FEMS Microbiol Rev 36:862–876. 10.1111/j.1574-6976.2011.00313.x 22091965

[34] Watve MG, Tickoo R, Jog MM, Bhole BD (2001) How many antibiotics are produced by the genus Streptomyces? Arch Microbiol 176:386–390. 10.1007/s002030100345 11702082

[35] Omura S, Ikeda H, Ishikawa J, Hanamoto A, Takahashi C, Shinose M, et al (2001) Genome sequence of an industrial microorganism Streptomyces avermitilis: deducing the ability of producing secondary metabolites. Proc Natl Acad Sci U S A 98:12215–12220. 10.1073/pnas.211433198 11572948 PMC59794

[36] Smanski MJ, Schlatter DC, Kinkel LL (2016) Leveraging ecological theory to guide natural product discovery. J Ind Microbiol Biotechnol 43:115–128. 10.1007/s10295-015-1683-9 26434742

[37] Kinkel LL, Bakker MG, Schlatter DC (2011) A Coevolutionary Framework for Managing Disease-Suppressive Soils. Annu Rev Phytopathol 49:47–67. 10.1146/annurev-phyto-072910-095232 21639781

[38] Cha J-Y, Han S, Hong H-J, Cho H, Kim D, Kwon SK, et al (2016) Microbial and biochemical basis of a Fusarium wilt-suppressive soil. ISME J 10:119–129. 10.1038/ismej.2015.95 26057845 PMC4681868

[39] Tomihama T, Nishi Y, Mori K, Shirao T, Iida T, Uzuhashi S, et al (2016) Rice Bran Amendment Suppresses Potato Common Scab by Increasing Antagonistic Bacterial Community Levels in the Rhizosphere. Phytopathology 106:719–728. 10.1094/PHYTO-12-15-0322-R 27050572

[40] Davelos AL, Kinkel LL, Samac DA (2004) Spatial Variation in Frequency and Intensity of Antibiotic Interactions among Streptomycetes from Prairie Soil. Appl Environ Microbiol 70:1051–1058. 10.1128/AEM.70.2.1051-1058.2004 14766588 PMC348876

[41] Davelos Baines AL, Xiao K, Kinkel LL (2007) Lack of correspondence between genetic and phenotypic groups amongst soil-borne streptomycetes. FEMS Microbiol Ecol 59:564–575. 10.1111/j.1574-6941.2006.00231.x 17381515

[42] R Core Team (2016) R: A language and environment for statistical computing. R Foundation for Statistical Computing, Vienna, Austria

[43] West DB (2001) Introduction to graph theory, 2nd ed Prentice Hall, Upper Saddle River, N.J

[44] Newman MEJ, Moore C, Watts DJ (2000) Mean-Field Solution of the Small-World Network Model. Phys Rev Lett 84:3201–3204. 10.1103/PhysRevLett.84.3201 11019047

[45] Csardi G, Nepusz T (2006) The igraph software package for complex network research. InterJournal Complex Systems:1695

[46] Benjamini Y, Hochberg Y (1995) Controlling the false discovery rate: a practical and powerful approach to multiple testing. J R Stat Soc 57:289–300

[47] Sarajlic A, Malod-Dognin N, Yaveroglu ON, Przulj N (2016) Graphlet-based Characterization of Directed Networks. Sci Rep 6:. 10.1038/srep35098 PMC5062067 27734973

[48] Yaveroğlu ÖN, Malod-Dognin N, Davis D, Levnajic Z, Janjic V, Karapandza R, et al (2015) Revealing the Hidden Language of Complex Networks. Sci Rep 4:. 10.1038/srep04547 PMC3971399 24686408

[49] D’Costa VM (2006) Sampling the Antibiotic Resistome. Science 311:374–377. 10.1126/science.1120800 16424339

[50] Leisner JJ, Jørgensen NOG, Middelboe M (2016) Predation and selection for antibiotic resistance in natural environments. Evol Appl 9:427–434. 10.1111/eva.12353 26989434 PMC4778110

[51] Vetsigian K (2017) Diverse modes of eco-evolutionary dynamics in communities of antibiotic-producing microorganisms. Nat Ecol Evol 1:0189 10.1038/s41559-017-0189

[52] Barabási A-L, Oltvai ZN (2004) Network biology: understanding the cell’s functional organization. Nat Rev Genet 5:101–113. 10.1038/nrg1272 14735121

[53] Schlatter DC, Kinkel LL (2014) Global biogeography of *Streptomyces* antibiotic inhibition, resistance, and resource use. FEMS Microbiol Ecol 88:386–397. 10.1111/1574-6941.12307 24580017

[54] Schlatter DC, Kinkel LL (2015) Do tradeoffs structure antibiotic inhibition, resistance, and resource use among soil-borne Streptomyces? BMC Evol Biol 15:. 10.1186/s12862-015-0470-6 PMC4570699 26370703

[55] Garbeva P, Tyc O, Remus-Emsermann MNP, van der Wal A, Vos M, Silby M, et al (2011) No Apparent Costs for Facultative Antibiotic Production by the Soil Bacterium Pseudomonas fluorescens Pf0-1. PLoS ONE 6:e27266 10.1371/journal.pone.0027266 22110622 PMC3217935

[56] Vaz Jauri P, Bakker MG, Salomon CE, Kinkel LL (2013) Subinhibitory Antibiotic Concentrations Mediate Nutrient Use and Competition among Soil Streptomyces. PLoS ONE 8:e81064 10.1371/journal.pone.0081064 24339897 PMC3855208

[57] Melnyk AH, Wong A, Kassen R (2015) The fitness costs of antibiotic resistance mutations. Evol Appl 8:273–283. 10.1111/eva.12196 25861385 PMC4380921

[58] Kirkup BC, Riley MA (2004) Antibiotic-mediated antagonism leads to a bacterial game of rock–paper–scissors in vivo. Nature 428:412–414. 10.1038/nature02429 15042087

[59] Kerr B, Riley MA, Feldman MW, Bohannan BJM (2002) Local dispersal promotes biodiversity in a real-life game of rock–paper–scissors. Nature 418:171–174. 10.1038/nature00823 12110887

[60] Ross-Gillespie A, Gardner A, Buckling A, West AS, Griffin AS (2009) Density Dependence and Cooporation: Theory and a Test With Bacteria. Evolution 63:2315–2325. 10.1111/j.1558-5646.2009.00723.x 19453724

[61] Wiggins BElizabeth, Kinkel LL (2005) Green manures and crop sequences influence alfalfa root rot and pathogen inhibitory activity among soil-borne streptomycetes. Plant Soil 268:271–283. 10.1007/s11104-004-0300-x 18943988

[62] Wiggins BE, Kinkel LL (2005) Green Manures and Crop Sequences Influence Potato Diseases and Pathogen Inhibitory Activity of Indigenous Streptomycetes. Phytopathology 95:178–185. 10.1094/PHYTO-95-0178 18943988

[63] Schlatter D, Kinkel L, Thomashow L, Weller D, Paulitz T (2017) Disease Suppressive Soils: New Insights from the Soil Microbiome. Phytopathology 107:1284–1297. 10.1094/PHYTO-03-17-0111-RVW 28650266

[64] Agler MT, Ruhe J, Kroll S, Morhenn C, Kim ST, Weigel D, et al (2016) Microbial Hub Taxa Link Host and Abiotic Factors to Plant Microbiome Variation. PLOS Biol 14:e1002352 10.1371/journal.pbio.1002352 26788878 PMC4720289

[65] Ojiambo PS, Scherm H (2006) Biological and Application-Oriented Factors Influencing Plant Disease Suppression by Biological Control: A Meta-Analytical Review. Phytopathology 96:1168–1174. 10.1094/PHYTO-96-1168 18943952

[66] Harcombe WR, Riehl WJ, Dukovski I, Granger BR, Betts A, Lang AH, et al (2014) Metabolic Resource Allocation in Individual Microbes Determines Ecosystem Interactions and Spatial Dynamics. Cell Rep 7:1104–1115. 10.1016/j.celrep.2014.03.070 24794435 PMC4097880

[67] Litchman E, Edwards KF, Klausmeier CA (2015) Microbial resource utilization traits and trade-offs: implications for community structure, functioning, and biogeochemical impacts at present and in the future. Front Microbiol 06: 10.3389/fmicb.2015.00254 PMC4389539 25904900

[68] Ponomarova O, Patil KR (2015) Metabolic interactions in microbial communities: untangling the Gordian knot. Curr Opin Microbiol 27:37–44. 10.1016/j.mib.2015.06.014 26207681

[69] Pilosof S, Porter MA, Pascual M, Kéfi S (2017) The multilayer nature of ecological networks. Nat Ecol Evol 1:0101 10.1038/s41559-017-0101 28812678

